# The Mechanism and Mediating Effect of the “Perception–Emotion–Behaviour” Chain of Tourists at World Natural Heritage Sites—A Case Study from Bayanbulak, China

**DOI:** 10.3390/ijerph182312531

**Published:** 2021-11-28

**Authors:** Qingliu Ren, Baoshi He, Xiaodong Chen, Jiali Han, Fang Han

**Affiliations:** 1 State Key Laboratory of Desert and Oasis Ecology, Xinjiang Institute of Ecology and Geography, Chinese Academy of Sciences, Urumqi 830000, China; renqingliu19@mails.ucas.ac.cn (Q.R.); hbs223@163.com (B.H.); chenxiaodong181@mails.ucas.ac.cn (X.C.); hanjiali21@mails.ucas.ac.cn (J.H.); 2 University of Chinese Academy of Sciences, Beijing 100049, China

**Keywords:** tourists, heritage gene perception, environmental knowledge perception, place attachment, pro-environmental behaviour intentions, Bayanbulak

## Abstract

The pro-environmental behaviour intentions (PEBIs) of tourists is a popular topic in tourism geography research. Visitors are important stakeholders in the development and conservation of World Natural Heritage sites (WNHs). Based on the perspective of the Mehrabian–Russell (M-R) theory, to advance our understanding of the transmission mechanism and mediation effect of the “perception–emotion–behaviour” chain of visitors at World Natural Heritage sites, we introduced two variables, namely heritage genes perception (HGP) and environmental knowledge perception (EKP), combined with place attachment (PA) and pro-environmental behaviour intentions (PEBIs), and scientifically constructed the conceptual model of the “EHPP model”, consisting of EKP, HGP, PA and PEBIs. Taking the Bayanbulak Heritage Site as an example, the EHPP model was fitted and tested using the structural equation model (SEM). The results show that: (1) the EHPP model is applied to fit the “cognitive–emotional–behaviour intentions” chain of visitors in WNHs and passed the empirical test; (2) there were positive and significant effects of EKP on HGP, and EKP indirectly affects PEBIs via HGP and PA; (3) place dependence (PD) had a significant and positive influence on place identity (PI); and (4) compliance with pro-environmental behaviour intentions (CPEBIs) had a direct positive influence on pro-environmental behaviour intentions (PPEBIs). The findings of this study provide empirical references for stimulating the pro-environmental behaviour intentions of tourists at World Natural Heritage sites.

## 1. Introduction

World Natural Heritage sites are natural landscapes of outstanding significance and universal value recognised by all humankind, inscribed on the World Heritage List, and recognised by UNESCO under the World Heritage Convention [1]. Because of their unique, rare natural appearance and outstanding academic value, World Natural Heritage sites have become unique and irreplaceable treasures shared by people all over the world [2]. With the popularity of mass tourism and the promotion of ecotourism, as an international brand with world-class natural landscapes, these sites often become preferred destinations for tourists, yet these areas are often ecologically fragile and sensitive. The behaviour intentions of tourists have a direct impact on the conservation and management of World Natural Heritage sites and any inappropriate behaviour intentions may lead to irreversible damage. In the process of developing tourism for the civilisation and popularisation of World Natural Heritage values, tourist behaviour intentions are seen as a precondition for sustainable heritage tourism. This sustainability will be realised if tourists’ cognition of the World Natural Heritage value and environmental knowledge is considered and integrated into the tourism development approach and if the key factors and driving paths of the pro-environmental behaviour intentions of tourists are explored.

At present, the existing tourism studies on World Natural Heritage Sites focus on sustainable tourism [3], tourism development [4], tourism attractiveness [5], tourism lands [6], tourism space reconstruction [7], and other aspects of heritage tourism development; they also work on the satisfaction of local communities [8], residents’ subjective well-being [9], tourist satisfaction [10], as well as tourist and other stakeholder perceptions of heritage tourism [11]. Sifeng Nian et al. took the perspective of attractions with outstanding universal value (OUV) and explored the mechanisms and influence of tourist heritage protection [12]. Norzaini Azman et al. believed that education plays a major role in shaping human behaviour intentions, and one of the key success factors for sustainable conservation is the level of appreciation and awareness of the heritage value of resources by stakeholders [13]. Chang Wang et al. explored the moderating role of environmental interpretations in the sightseeing place on the relationship between tourists’ responsible environmental behaviour intentions (REBIs) and REB [14]. Based on previous research and regional characteristics, this paper attempts to introduce elements of integrity, authenticity and core landscape in visitor perception, bring self-directed education and other-directed education in environmental education at World Natural Heritage Sites. What is the interaction among the perception of World Natural Heritage value, the perception of environment education, and the place attachment and the pro-environmental behaviour intentions of tourists? How can a theoretical model be established to explore their interrelationships? What are the mechanisms and mediation effects of these four interactions? There are few studies on these issues in China. This research focuses on the practical problem of “how to stimulate the pro-environmental behaviour intentions of tourists at World Natural Heritage sites” and develops the EHPP model of the relationship between environmental knowledge perception (EKP), heritage genes perception (HGP), place attachment (PA) and pro-environmental behaviour intentions (PEBIs). This research acknowledges the theoretical and practical gap and aims to fill the gap by providing a more comprehensive framework for understanding the formation of pro-environment behaviour intentions, and address the issue of guiding the management of pro-environmental behaviour intentions of visitors at World Natural Heritage sites. This is important practical guidance to reduce the ecological and environmental problems caused by tourists in the process of visiting, to strictly protect the integrity and authenticity of the World Natural Heritage sites, and to realise the sustainable development of humans and nature.

### 1.1. Theoretical Basis

In 1974, environmental psychologists Mehrabian and Russell proposed the Mehrabian–Russell (M-R) model, also called the stimulus–organism–response (SOR) theory [15]. The model includes three major parts: environmental stimulus (ES), affective state (AS), and approach and avoidance behaviour intentions (AABs). It states that when an individual encounters a stimulus (S), they develop internal states (O), which in turn dictate their response (R) [16]. This is for the component of the environment external to the individual to provide the stimuli which are then internally processed by the organism. Specifically, stimuli develop individuals’ cognitive and emotional states, which in turn determine behaviour intention responses of approach or avoidance [17,18]. Applied to explore the mechanism of the tourist “perception–emotion–behaviour” action, the heritage genes perception and environmental knowledge perception of tourists can be regarded as the first building block of the M-R model-ES; place attachment can be seen as the second building block of the M-R model-AS; pro-environmental behaviour intentions can be considered the third building block of the M-R model-AAB. In general, tourists gain knowledge through environmental education, recognise the value of World Natural Heritage sites, and then develop emotional feelings such as place dependence (PD) and place identity (PI), which finally act to produce compliance pro-environmental behaviour intentions (CPEBIs) and positive pro-environmental behaviour intentions (PPEBIs).

### 1.2. Concept Definition

Heritage genes perception: existing research on visitor perception mostly considers some externally demonstrated travellers’ perceptions of experience quality [19], tourists’ perception of crowding [20], the risk perception attitude [21], etc. Aesthetic dimensions of tourists in the context of both nature-based and urban tourist destinations were proposed in a previous study [22]. Fyhri, Jacobsen, and Tømmervik in particular examined international visitors’ landscape perceptions in a coastal area [23]. The tourism development of World Natural Heritage sites should give more consideration to resource conservation and incorporate integrity, authenticity, and aesthetic landscapes into the heritage perception system. Authenticity and integrity are important guiding principles for world heritage conservation, and a yardstick for measuring the value of natural heritage fundamental to ensuring the sustainability of heritage sites. Ecosystem integrity initially refers to the preservation of biome integrity, stability, aesthetics, etc. [24]. Existing studies explain that integrity means that the ecosystem has the biodiversity and ecological processes contained in the natural habitat of the region, and means a term that is often used to describe the state of ecosystems subjected to anthropogenic pressures and is usually closely aligned to the literal definition of integrity: being whole or unimpaired [25]. Ecosystem health, stability, and sustainability are also important expressions of integrity [26]. Ecosystem authenticity refers to pristine natural areas that have not been significantly disturbed by humans and where ecosystems and ecological processes are in a high-quality natural state [27,28]. In addition, the core landscape resources are the most intuitive and visually stunning high-quality aesthetic landscapes that the World Natural Heritage site offers to visitors. Heritage genes perception refers to tourists’ comprehensive feelings about the integrity, authenticity, and core landscape resources of World Natural Heritage sites.

Environmental knowledge perception: learning experiences that happen in an informal and carefree setting (such as tourism activities) tend to educate people more than formal education settings such as the school environment [29]. Environmental education is an important informal way to generate and disseminate environmental knowledge, aimed to influence the degree of visitors’ environmental awareness through recreational activities during their vacation [30]. Environmental education is generally divided into self-directed education (e.g., visual interpretation [31], environmental art [32]) and other-directed education (e.g., tour guides’ performance [33], formal tour guiding operations [34]). Self-directed education helps visitors understand landscape resources, preserve natural resources, and strengthen visitors’ environmental awareness through the medium of interpretive signs, publications, multimedia interpretive systems, and visitor centres. Other-directed education means that visitors are educated under the introduction, guidance or experience of others, such as tour guides and interpreters. environmental knowledge perception is the sense of education obtained by the visitor’s interaction with the natural environment, mainly for the protection of flora and fauna, under the dual system of self-guided education and other-guided education.

Place attachment: Taylor and Shumaker were the first to clarify the concept of place attachment, defining it as the emotional connection between people and places, emphasising the positive emotional attachment that people develop psychologically to a place [35]. In terms of place attachment dimensions, the two-dimensional scale developed by Williams, which includes place dependence and place identity, is a typical example [36]. Place attachment refers to the tourist’s perception of the functional attachment and environmental uniqueness of a specific resort area [36]. Place identity is the expression of a tourist’s self-identification with a specific resort area through a series of conscious and unconscious memories, opinions, emotions, attitudes, evaluations, preferences, and behaviour intentions [37].

Pro-environmental behaviour intentions (PEBIs): although individual scholars have sorted the concepts of environmentally responsible behaviour intentions, pro-environmental behaviour intentions, environmentally concerned behaviour intentions, environmentally significant behaviour intentions, and sustainable behaviour intentions [38], some scholars have argued that the above terms are used interchangeably [39,40], and in fact, some literature directly treats the concepts of environmentally responsible behaviour intentions and pro-environmental behaviour intentions as equivalent concepts [41,42]. Pro-environmental behaviour intentions (PEBIs) are behaviour intentions that consciously minimise the negative impact of one’s actions on the natural and built worlds [43,44]. Environmentally responsible behaviour intentions (PEBIs) refer to an individual’s determination of the subjective probability of their performance of certain environmental behaviour intentions [14,45]. This not only includes environmentally responsible behaviour intentions in the private sphere, such as not littering, but also in the public sphere, such as picking up litter, reminding and warning fellow tourists to properly behave in a tourist locations [46]. The environmentally responsible behaviour intentions of tourists are an important driver of the sustainable use of resources in tourist locations. Environmentally responsible behaviour intentions include compliance with environmentally responsible behaviour intentions (CERBIs) and positive environmentally responsible behaviour intentions (PERBIs).

### 1.3. Relationship Interpretation

Research related to environmental knowledge perception: environmental education is carried out in the form of environmental interpretation and environmental activities during the tourist process. Arrival briefings are also an effective interpretation strategy in tourist destinations [47]. Tourism interpretation is an important approach to assisting tourists in obtaining the authentic values of heritage destinations so that the effectiveness of tourism interpretation in delivering natural and cultural values becomes of significance [48]. Tourism interpretation in the sightseeing place positively moderates the relationship between tourists’ responsible environmental behaviour intentions (REBIs) and responsible environmental behaviour intentions (REBIs) [14]. Informal education activities can act as triggers for environmental awareness and behaviour intentions in tourists, providing them with the tools, knowledge, and motivation to critically discern what is and is not environmentally friendly. Some studies have shown that tourists’ PA has a significant positive effect on perceived interpretation service quality [49]. The above experience shows that environmental education can also make tourists fully aware of the integrity and authenticity of World Natural Heritage sites, both in theory and in practice.

Research related to heritage genes perception: the “cognitive–emotional” theory suggests that people first recognise what is happening around them and then develop the corresponding emotions; cognition is a necessary but not a sufficient condition for the development of emotions. Baloglu and McCleary constructed a destination image model based on a literature review, and empirical studies proved that cognitive images have a positive effect on emotional images [50]. Likewise, the research showed that two factors of cognitive images (attraction and comfort) have a positive effect on emotional images [51]. Visitor perceptions that have been shown to have direct or indirect effects on visitor behaviour intentions include perceived value [52], destination risk perception [53], and food taste perception in tourism destinations [54]. In the case of World Natural Heritage sites, the mechanism of action between visitors’ perception of heritage genes and pro-environmental behaviour intentions have not been addressed. Can tourists’ perception of genes of World Natural Heritage sites bring emotional attachment and identification? Is it possible to restrain tourists’ harmful behaviour intentions?

Research related to place attachment: Kaltenborn first proposed the role of local attachment in promoting pro-environmental behaviour intentions when studying the environmental protection behaviour intentions of local residents in Norway [55]. Place attachment not only directly contributes to pro-environmental behaviour intentions, but also mediates between environmental knowledge and pro-environmental behaviour intentions. In terms of the relationship between place dependence and place identity, most scholars believe the following: visitors generally develop place dependence before further forming place identity [56]; that place identity has a significant positive effect on all dimensions of pro-environmental behaviour intentions, while place dependence shows insignificance [42]; and that place identity plays a mediating role in the analysis of the relationship between place dependence and pro-environmental behaviour intentions [57].

Research related to pro-environmental behaviour intentions: specific environmentally sustainable tourist behaviour intentions have distinctly different drivers [58]. Attitude, subjective norms, perceived behaviour intentional control, and incentive measures significantly affect behaviour intentions; environmental theory knowledge and environmental practice knowledge have had indirect effects on behaviour intentions via the mediator of attitude towards the behaviour intentions [59]. Furthermore, place attachment, conceptualised as community attachment and connectedness to nature, was found to be a significant predictor of pro-environmental behaviour intentions in some studies [60,61].

## 2. Materials and Method

### 2.1. Hypotheses and Conceptual Model

**Figure 1:**
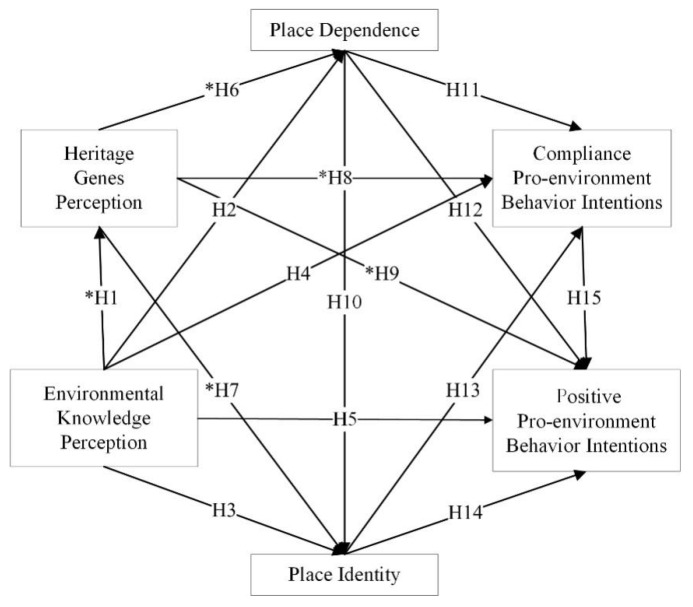
EHPP conceptual model. Note: * indicates hypotheses empirically tested for the first time using the structural equation model.

In summary, a very important and urgent question is: What are the correlations and mechanisms between the HGP, EKP, PA, and PEBIs of visitors to World Heritage Natural sites? On the basis of the above literature review, the study proposes an EHPP conceptual model (Figure 1), consisting of six variables: HGP, EKP, PD, PI, CPEBIs, and PPEBIs. EHPP has been defined as a conceptual model in which visitors are stimulated by environmental knowledge and heritage genetic landscapes that promote the emotions of place dependence and place identity. These emotions can guide their behaviour intentions. The goal of the model is to discover the linkages and mechanisms among variables and then provide theoretical guidance and empirical reference for the chain of the perception–behaviour–intentions relationships of tourists at World Natural Heritage sites.

Based on the above discussion, we proposed the following hypotheses:

**Hypothesis** **1** **(H1).** *Tourists’ EKP has a significant positive impact on HGP.*

**Hypothesis** **2** **(H2).** *Tourists’ EKP has a significant positive impact on PD.*

**Hypothesis** **3** **(H3).** *Tourists’ EKP has a significant positive impact on PI.*

**Hypothesis** **4** **(H4).** *Tourists’ EKP has a significant positive impact on CPEBIs.*

**Hypothesis** **5** **(H5).** *Tourists’ EKP has a significant positive impact on PPEBIs.*

**Hypothesis** **6** **(H6).** *Tourists’ HGP has a significant positive impact on PD.*

**Hypothesis** **7** **(H7).** *Tourists’ HGP has a significant positive impact on PI.*

**Hypothesis** **8** **(H8).** *Tourists’ HGP has a significant positive impact on CPEBIs.*

**Hypothesis** **9** **(H9).** *Tourists’ HGP has a significant positive impact on PPEBIs.*

**Hypothesis** **10** **(H10).** *Tourists’ PD has a significant positive impact on PI.*

**Hypothesis** **11** **(H11).** *Tourists’ PD has a significant positive impact on CPEBIs.*

**Hypothesis** **12** **(H12).** *Tourists’ PD has a significant positive impact on PPEBIs.*

**Hypothesis** **13** **(H13).** *Tourists’ PI has a significant positive impact on CPEBIs.*

**Hypothesis** **14** **(H14).** *Tourists’ PI has a significant positive impact on PPEBIs.*

**Hypothesis** **15** **(H15).** *Tourists’ CPEBIs have a significant positive impact on PPEBIs.*

### 2.2. Study Area and Methodology

#### 2.2.1. Study Area Overview

**Figure 2:**
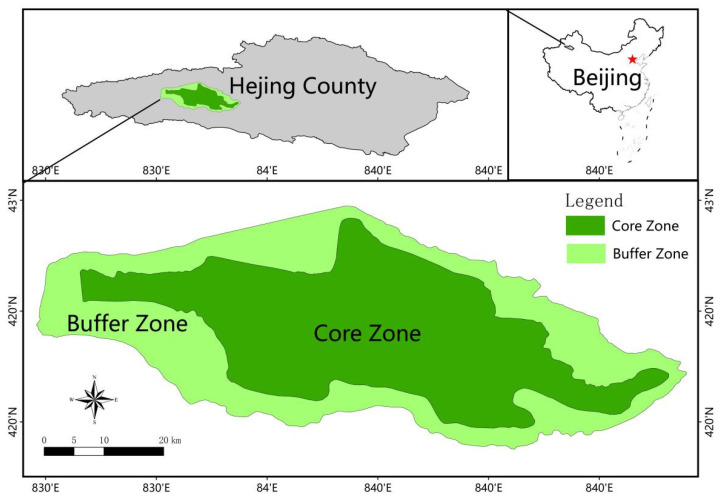
Bayanbulak Heritage Site geographical location (Source: GS(2016)2893).

The Bayanbulak Heritage Site is located in Hejing County, Bayingoleng Mongol Autonomous Prefecture, Xinjiang, China, 83°37′–84°22′ E, 42°20′–42°55′ N, covering a total area of 136,894 hectares (Figure 2). The Yurdus Basin and the Kaidu River Wetland in the middle of the Tianshan Mountains are the main landscape objects, with *Cygnus cygnus*, *Cygnus columbianus* and their habitat ecosystems as the main conservation objects. The heritage site is a typical representative of the large intermountain basin in the Tianshan Mountains; a typical representative of the alpine wetland ecosystem in the temperate arid zone; the most typical representative of the beauty of the landscape of the Tianshan River Quagmire; the largest breeding site of swans in China; and the southernmost limit of wild swan breeding in the world. It was listed as a World Natural Heritage project in 2013 with Xinjiang Tianshan Series Heritage.

In recent years, tourist activities have placed certain pressures on the natural resources and ecological environment of heritage sites. According to our survey, the daily visitor volume of the Bayanbulak Heritage Site can reach 10,000 during peak season, leading to the environmental capacity becoming saturated and overloaded due to the overconcentration of tourists, and the uncivilised behaviour intentions of some tourists has caused indelible damage to the ecological environment and even threatened wildlife migration and habitat conservation at the heritage site. This study took the Bayanbulak Heritage Site as a case study site, which is of practical significance and a guide to study the pro-environmental behaviour intentions of tourists at World Natural Heritage sites.

#### 2.2.2. Questionnaire Design

The questionnaire used for the survey consists of two parts. The first is related to the economic and social characteristics of tourists, including demographic characteristics (gender, age, ethnicity, occupation, education level, monthly personal income, place of residence) and other information (place of origin, number of trips, travel route, knowledge, purpose of visit, and tourist facilities noted), thus identifying the social attributes of tourists. The second part is a survey of visitors’ perceptions, emotions and behaviour intentions. This part involves the measurement of six latent variables: the HGP, EKP, PD, PI, CPEBIs, and PEBIs of visitors to Natural World Heritage sites. To minimise data overbias in the use of structural equation modelling, SEM, a five-point Likert scale was used for the research scale in the questionnaire, corresponding to measures 1–5 (1 = strongly disagree; 5 = strongly agree).

HGP consisted of 10 questions and was designed from the perspective of visitors’ perceptions of the integrity, authenticity, and core aesthetic landscape in World Natural Heritage sites; EKP consists of four questions, which are mainly based on self-directed education and other-directed education; PA had a total of 6 items, including 3 items describing PD and 3 items describing PI, referenced from Stylos Nikolaos [62]. There are seven questions on PEBIs, including 3 questions describing CPEBIs and 4 questions describing PPEBIs, as drawn from Haywantee Ramkissoon [63].

#### 2.2.3. Questionnaire Research

To ensure the quality of the questionnaire answers collected, the questionnaire distributors were all graduate students. Before conducting the questionnaire distribution, the main purpose of the survey and the survey requirements were explained to them so that they could be familiar with the research topic and master certain questionnaire distribution skills. A presurvey was conducted before the formal questionnaire, and the questions in the questionnaire were revised through feedback. Because of the global pandemic, almost all the visitors were from China. The formal research was conducted from 24 July 2021 to 30 July 2021, involving Chinese tourists, and the location of the research was in nine curves and eighteen bends of the site. Interviews and questionnaires were conducted with tourists by random interception. Four hundred questionnaires were distributed and 342 questionnaires were collected, resulting in an 85.5% recovery rate. After eliminating invalid questionnaires (not completed or not objective), 307 valid questionnaires were obtained, the efficiency rate was 90%. The data requirements of the structural equation model were reached (*n* ≥ 50r^2^ − 450r + 1100), where *n* is the sample size and r is the ratio of indicators to latent variables [64].

#### 2.2.4. Analysis Method

This study adopted factor analysis (FA) which is a widely used method for both reducing the dimensionality of variables and classifying them. The essence of FA is to extract fewer, uncorrelated, abstract composite indicators, i.e., factors, from multiple measured original variables [65]. SPSS 25.0 software was used to conduct an exploratory factor analysis on 30 items, and factor analysis was performed by the Kaiser standardised maximum variance orthogonal rotation method to extract principal components with eigenvalues greater than 1. The factor loading intercept point was 0.5, the items that were loaded below 0.5 on any factor or more than 0.4.

The structural equation model (SEM) [66] in Amos based on maximum likelihood estimates is also applied. On the one hand, structural equation modelling can deal with both latent variables and their indicators; on the other hand, when testing hypothesised relationships in the study, both the independent and dependent variables are latent variables with measurement errors. The structural equation model considers the presence of errors in the model fitting [67]. SEM includes the measurement models and the structural models. The measurement model can reveal the relationship between measurable variables and latent variables, and the structural model can reveal the relationship between latent variables and latent variables [68].

The combined use of the above methods aims to verify the relationships among HGP, EKP, PA, and PEBIs.

## 3. Results

### 3.1. Descriptive Statistical Analysis

As shown in Table 1, the demographic characteristics of tourists include nine attributes: gender, age, ethnicity, education level, occupation, monthly income, place of residence, number of visits and mode of travel. Among the respondents, 52.1% were male and 47.9% were female. The largest age groups were 26–46 years old (50.2%), with ages 18–25 years accounting for 24.8%, 20.1% were between 47 and 60 years old and 4.9% were over 60 years old; 87.3% of responses were Han Chinese, the rest consisted of Uighur (2.9%); Hui (3.6%); Mongolian (2.9); and others (3.3). The majority of respondents were university students, representing 61.6%—17.6% of them were postgraduate and above. Among the respondents, 16.6% and 4.2% were educated to only a junior and secondary high school level, respectively. With regard to occupation characteristics, 37.8% of the visitors worked for enterprises or institutions. The largest responses of annual income were between CNY 5001 and CNY 10,000, whilst 22.5% responses showed an annual income below CNY 3000, and 21.5% of respondents had an annual income over CNY 10,001; finally, 32.9% of visitors earned in the range of CNY 5001–10,000/year.

In addition to the demographic characteristics of visitors, 75.6% of the tourists were visiting for the first time, 70.4% of the tourists were from other provinces, and 62.5% of the tourists drove themselves, A total of 54.1% of the tourists learned about the Bayanbulak Heritage Site through online channels such as WeChat, Weibo, and short videos; and 90.6% of the tourists considered the ecotourism program to be the most attractive; 85.7% of visitors noticed the ecotourism trail as an infrastructure.

### 3.2. Structural Equation Model

To ensure the stability and reliability of the scale, the reliability and validity of the sample containing 27 question items were tested before conducting the sample analysis. The internal consistency test was conducted by calculating the Cronbach’s alpha (α) value of the scale (greater than 0.7), and the results showed that the scale had a high combination reliability (CR) with CR = 0.919. Meanwhile, Kaiser–Meyer–Olkin (KMO) and Bartlett’s spherical tests were used to explore the suitability of the sample data for factor analysis, and the results showed that the KMO value was 0.92, Bartlett’s spherical test chi-square value was 6688.782, the significance level was 0.000 < 0.001, and the statistical tests for the validity of the 27-question items were significant, verifying that the data were suitable for factor analysis [69].

#### 3.2.1. Exploratory Factor Analysis

The results of the factor analysis showed that a total of 5 items that did not meet the conditions were deleted, leaving 22 items. The results showed that 5principal components were extracted and explained 68.007% of the total variance, which exceeded the minimum criterion of 60% variance contribution (Table 2). PA always exists in the form of two latent variables, PD and PI, in most of the literature. To ensure the fitness of the model and combine the results of principal component analysis, the 22 items were divided into 6 latent variables: HGP, EKP, PD, PI, CPEBIs, and PPEBIs. To guarantee the quality of the scale, the six extracted latent variables were separately tested for structural validity and internal consistency. The results are shown in Table 3. Except for the question “I like this place better than other scenic spots”, which had a factor loading less than 0.5—0.484—the factor loadings of all other questions were greater than 0.7. The internal consistency coefficient Cronbach’s α values were greater than 0.8, indicating that each scale had good reliability; apart from PD, the KMO coefficients of the other five latent variables were greater than 0.7, showing the high validity of the sample data and the significant consistency and validity of the measurement results [70].

#### 3.2.2. Validation Factor Analysis

1. Measurement Model Check

① Model suitability

Validation factor analysis was conducted using AMOS 26.0 and a first-order validation measurement model was developed based on the results of exploratory factor analysis. First, structural validity tests were conducted. The sample number of this project was 307, and χ2/df can better reflect the fitness than χ2. Usually, χ2/df below 5 is a good fit (below 3 is better) [69]. The structural equation model was fitted and tested using the criteria proposed by Ming-Lung Wu, and the model fit was good, but indicators such as GFI and NFI did not meet the requirements of a good model, and the goodness of model fit needed to be improved.

The model needs further revision due to unsatisfactory first adaptation results. The modification indices (MI) term output from the AMOS fitting results can be used as a correction reference. The MI coefficient correction method was used to release the error terms of the measured variables under the same latent variable and increase the covariance relationship between the error terms. The main approach was to add residual term correlations one by one for paths with large MI values. Specifically, since there is a certain covariance between the question item “The interpreter’s presentation helped me to learn some knowledge” and the question item “Tour guide’s presentation helped me to learn some knowledge”, it is reasonable to increase the covariance of the error term. After one correction of MI coefficients, the model fitness reached the optimal effect (Table 4).

② Combination validity

After a good validation model was obtained, the reliability and validity of each dimension were separately tested to verify the applicability of the model. The results of the analysis are shown in Table 5. The standardised factor loadings (Std) of the questionnaire items ranged from 0.625 to 0.954 except for PD3, all of which met the criterion of 0.5 and were significant (*p* value = 0.000); the CR of each latent variable was greater than 0.7; the average of variance extracted (AVE) reached the standard of 0.5—the higher the AVE, the higher the convergence effect.

③ Distinct validity

The distinct validity among the latent variables was examined and the results are shown in Table 6. It is generally agreed that the squared correlation coefficients between each latent variable are smaller than the AVE of each latent variable, indicating a better distinct validity between the latent variables. The results showed that the square root of AVE for each latent variable was higher than the correlation coefficient of that latent variable with other latent variables, except for the correlation coefficient values of PD and PI (0.852), which were higher than PI’ s AVE (0.668). In conclusion, the distinct validity between latent variables was better.

Therefore, the reliability and validity tests of these sample data met the acceptability criteria and further hypothesis testing could be performed.

2. Structural Model Check

**Figure 3:**
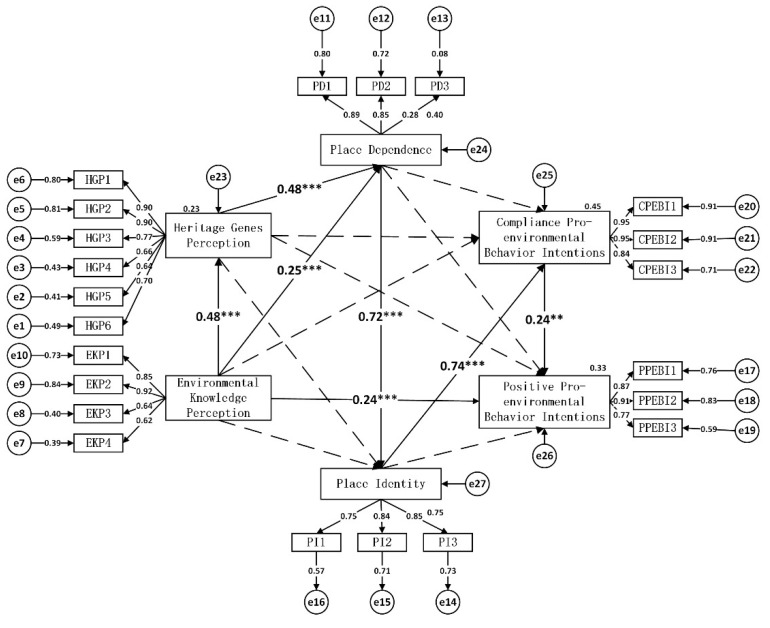
Results of the hypotheses test. ** *p* < 0.01, *** *p* < 0.001.

The results showed that EKP had a significant positive effect on HGP, PD, and PPEBIs (H1, H2, and H5 held) (see Figure 3, Table 7). In other words, the environmental education that tourists receive during their visit will not only give them a deeper understanding of the scientific value of the heritage site and make them feel dependent on it but also directly inspire them to develop PPEBIs and gradually transform them from individualists to ecologists. There was no significant effect of EKP on either PI or CPEBIs (H3 and H4 did not hold). The reason for this is that there is still a gap between the Bayanbulak Heritage Site and mature World Natural Heritage sites abroad in terms of environmental education. As outsiders, tourists have difficulty forming local identity at the beginning of the development of local dependency, fail to form CPEBIs, and still need self-guided and other-guided education to guide and restrain them in the future. The originality and highly aesthetic core landscapes of World Natural Heritage sites can create place-dependent emotions in visitors.

The effects of HGP on PI, CPEBIs and PPEBIs were not significant (H7, H8, and H9 did not hold). PD had a significant positive effect on PI (H10 held), validating the positive effect of PD on PI proposed by most scholars. The effect of PD on both CPEBIs and PPEBIs was not significant (H11 and H12 did not hold). Since the factor loading of the third variable measuring PD was low and insufficient to explain it, it may have affected the measurement of the relationship between PD and PPEBIs. PI had a significant positive effect on CPEBIs (H13 held). Personal emotional identity plays a major role in protecting visitors in nature and getting closer to nature. Once the identity bond is created, visitors themselves will create a self-binding force, making the willingness to limit self-unfriendly behaviour intentions stronger. The effect of local identity on PPEBIs was not significant (H14 did not hold), and CPEBIs had a significant positive effect on PPEBIs (H15 held).

3. Mediation Effect Check

The mediation effects between EKP, PA, and PEBIs were further explored with the hypothesis testing results. This paper used the bootstrapping-mediated effects’ test proposed by Hayes [71] to test the mediating effects. The SEM was drawn by AMOS 24.0, a 95% confidence interval was set, and 5000 bootstrapping samples were taken to test for mediating effects using the bootstrapping ML method. The following two steps need to be completed to test the mediation effect. First, if the overall effect t-value >1.96 and the value in the reliance interval in the bias-corrected method and percentile method is not 0, it means that the overall effect in the path is significant and the mediation effect may exist, and if not, the mediation effect does not exist. Second, related to the indicators of indirect and direct effects, the same requirement is a t-value >1.96 and the value in the reliance interval in the bias-corrected method and percentile method is not 0. If both the indirect and direct effects satisfy the condition, it means that there is a partial mediating effect between the variables, or if the indirect effect satisfies the condition and the direct effect does not satisfy the condition, it means that there is a full mediating effect between the variables.

The mediation effect results can be found in Table 8 below. ① The overall, direct and indirect effects in the “EKP→HGP→PD” (path 1) pathway are significant, and the HGP partially mediates the role between EKP and PD. ② The influence of EKP on PI has two paths, including “EKP→PD→PI” (path 2) and “EKP→HGP→PD→PI” (path 3). Because the direct effects of the two paths are not significant, HGP and PD play a fully mediating role between EKP and PI, and the coefficients of the two paths are approximately equal. ③ The influence of EKP on CPEBIs has two pathways: “EKP→PD→PI→CPEBIs” (path 4) and “EKP→HGP→PD→PI→CPEBIs” (path 5). Similarly, because the direct effects of the two pathways are not significant, HGP, PD and PI play a fully mediated role between EKP and CPEBIs, and the coefficients of the two pathways are approximated. ④ There are two paths of EKP on PPEBIs: “EKP→PD→PI→CPEBIs→PPEBIs” (path 6) and “EKP→HGP→PD→PI→CPEBIs→ PPEBIs” (path 7). The overall, direct, and indirect effects in the “EKP→PPEBIs” pathways were significant, while HGP, PD, PI, and CPEBIs mediated between EKP and PPEBIs with very small coefficients.

## 4. Discussion

This study focuses on the pro-environmental behaviour intentions of tourists at World Natural Heritage sites and proposes an EHPP model based on the M-R theoretical framework. Overall, two major findings were identified in the current study, explained in detail below.

The EHPP model was applied to fit the “cognitive–emotional–behaviour” chain of visitors at World Natural Heritage sites and was empirically tested. Consistent with the results of previous studies, the acquisition of environmental knowledge not only directly influences the pro-environmental behaviour intentions of tourists, but also stimulates the dual emotions of place dependence and place identity by improving tourists’ perceptions of heritage genes and the external core landscape, which leads to pro-environmental behaviour intentions [72]. This reflects the important role of self-directed and other-directed environmental education in restraining visitor behaviour intentions, and shows that the mass route of environmental knowledge and heritage values is also the most important link in the conservation of the integrity and authenticity of World Natural Heritage sites. This also reflects the fact that place attachment is a necessary cue to influence tourist’ pro-environmental behaviour intentions. The practice of environmental education and the generation of local emotions are both important ways of nurturing visitors’ pro-environmental behaviour intentions.

This paper further explored the mediating effects between environmental knowledge perception and place attachment, and between environmental knowledge perception and pro-environmental behaviour intentions. Heritage genes perception plays a partial mediating role in environmental knowledge perception and place dependence and account for 48.1% of the total effect, indicating that environmental knowledge can directly generate place dependence emotions in visitors, and indirectly contribute to it through heritage genes perception. There is no significant direct effect between environmental knowledge perception and place identity, and only a significant mediating effect. This not only validated the accepted result that tourists develop place dependence before place identity [73], but also verified the mediating role of heritage genes perception and place dependence. There was also no significant direct effect of environmental knowledge perception and compliance with pro-environmental behaviour intentions, and heritage genes perception and place attachment played an important role in motivating visitors to restrict their own behaviour intentions. For the production of positive pro-environmental behaviour intentions, this mainly occurs through the direct facilitation of environmental knowledge perception which means that self-directed and other-directed environmental education plays a crucial role in guiding positive pro-environmental behaviour intentions while the resultant coefficient of the mediating role is small and insignificant.

### 4.1. Theoretical Implications

This study contributes to the existing literature on several fronts. In spite of the existing literature having explored the mechanisms of visitor behaviour intentions, no research had been conducted to examine the role of heritage genes perception on tourist’ emotion and behaviour intentions at World Natural Heritage sites. This study addressed this gap in the literature by identifying the role that environmental knowledge perception and heritage genes perception play in the tour through the proposed EHPP model. Environmental knowledge perception is the precursor to place attachment and pro-environmental behaviour intentions, with heritage genes’ perception playing a crucial intermediary role. Furthermore, place dependence is the antecedent of place identity, place identity is the result of place dependence. The fact that visitors generate place dependence before contributing to place identity, also validates most of the literature. Thirdly, compliance with pro-environmental behaviour intentions is a direct result of place attachment. During the tour, only by developing emotional dependence and identity can we better constrain our behaviour intentions. environmental knowledge perception and place attachment together contribute to the creation of positive pro-environmental behaviour intentions.

### 4.2. Practical Implications

The practical implications of the science education system should be noted for Natural Heritage sites’ planning and marketing. The management should focus on protection, designing, and advertising the integrity and authenticity of natural resources to visitors. For example, the management centre could use conventional ways (e.g., ecological indicator, interpretation centre, and relevant signage) or upcoming technologies (e.g., mini programs of audio interpretation, human–computer interaction, and virtual reality) to facilitate tourists’ absorption of environmental knowledge perception and HGP.

### 4.3. Limitations

Although our study was by no means exploratory in nature, it does mark the first time that HGP has been considered. Several limitations provide potential avenues for future research. First, this paper concentrated on quantitative analysis, which can be combined with qualitative research to analyse the formation mechanism of PEBIs in depth in the future. Second, this paper explored the introduction of heritage genes perception and environmental knowledge perception into the driving antecedents of tourists’ pro-environmental behaviour intentions. Although the scale design and empirical evidence show that the model fits well, whether the scale has wide applicability to other World Natural Heritage sites needs to be verified by adding empirical case studies.

## 5. Conclusions

The EHPP model vas proposed to investigate the relevant relationships among environmental knowledge perception (EKP), heritage genes perception (HGP), place dependence (PD), place identity (PI), compliance pro-environmental behaviour intentions (CPEBIs), and positive pro-environmental behaviour intentions (PPEBIs). The Bayanbulak Heritage Site in Hejing County, Xinjiang, China, was chosen as location for the survey. This research provides strong support for understanding the relationship between visitors and nature at World Natural Heritage sites. Results suggest that EKP is the precursor to PA and PEBIs, with HGP playing a crucial intermediary role. Place attachment can restrain our own behaviour intentions, and environmental knowledge education is the key to guide others’ ecological behaviour intentions. Overall, this research provides the first study on introducing potential variables of self-guided education, other-guided education, integrity, and authenticity of World Natural Heritage sites. It illustrates the mechanism and mediating effect of the “perception–emotion–behaviour” chain of tourists at World Natural Heritage sites.

## Figures and Tables

**Table 1:** Descriptive statistics of the demographic characteristics of the samples (*n* = 307).

Demographic	Type	Percentage	Demographic	Type	Percentage
Sex	Male	52.1	Occupation	Tourism-related staff	6.2
	Female	47.9		Enterprise and business Unit staff	37.8
Age	18–25	24.8		Private owners	10.7
	26–46	50.2		Freelance	7.2
	47–60	20.2		Retirees	6.5
	>60	4.9		Workers	3.3
Ethnicity	Han	87.3		Students	19.9
	Uighur	2.9		Other	8.5
	Hui	3.6	Place of residence	Bayingol Mongolian Autonomous Prefecture	8.8
	Mongolian	2.9		Xinjiang Uygur Autonomous Region	20.8
	Other	3.3		Other provinces	70.4
Education level	Junior high school	4.2	Frequency of interaction	First time	75.6
	Secondary education	16.6		Second time	10.4
	Bachelor’s degree	61.6		Three times or more	14.0
	Master’s degree or above	17.6	Mode of travel	Travel agency	14.7
Average monthly income (CNY)	≤3000	22.5		Group trips	6.5
	3001–5000	23.1		Self-driving travel	62.5
	5001–10,000	32.9		Travel by car	11.7
	≥10,001	21.5		Other	4.6

**Table 2:** Total variance of interpretation.

Components	Initial Eigenvalue			Sum of Squared Rotating Loads		
	Total	Percentage of Variance	Cumulative Percentage	Total	Percentage of Variance	Cumulative Percentage
1	10.859	40.218	40.218	4.636	17.170	17.170
2	2.694	9.976	50.195	4.273	15.825	32.996
3	2.083	7.713	57.908	3.504	12.978	45.974
4	1.502	5.561	63.469	3.207	11.879	57.853
5	1.225	4.538	68.007	2.742	10.154	68.007
6	0.946	3.505	71.512			

**Table 3:** Exploratory factor analysis of the measurement project.

Factor Naming	Dimensions	Measurement Topics	Factor Loadings	KMO and Bartlett Test	Cronbach’s α
Heritage genes perception	Integrity	Integrity of grassland ecosystems (HGP1)	0.881	KMO = 0.859 Sig. = 0.000	0.890
		Integrity of wetland ecosystem (HGP2)	0.883		
		Integrity of the overall landscape (HGP3)	0.825		
	Authenticity	The natural landscape presents a natural state and a wilderness state, undisturbed by humans (HGP4)	0.731		
	Core landscape	Nine curves and eighteen bends (HGP5)	0.731		
		Alpine meadow landscape (HGP6)	0.780		
Environmental knowledge perception	Self-directed education	A variety of environmental interpretation signs and environmental protection markings are installed in the scenic area (EKP1)	0.836	KMO = 0.732 Sig. = 0.000	0.861
		I learned about environmental protection from the visitor centre, scenic guide signs and related banners (EKP2)	0.875		
	Other-directed education	The interpreter’s presentation helped me to learn some knowledge (EKP3)	0.840		
		Tour guide’s presentation helped me to learn some knowledge (EKP4)	0.829		
Place attachment	Place dependency	I feel like I will not forget about the beauty of sightseeing here (PD1)	0.910	KMO = 0.546 Sig. = 0.000	0.848
		I enjoy sightseeing, photography, horse riding and recreation here (PD2)	0.908		
		I like this place better than other scenic spots (PD3)	0.484		
	Place identity	This tour means a lot to me (PI1)	0.846	KMO = 0.719 Sig. = 0.000	0.848
		I agree that the site has high natural heritage value (PI2)	0.894		
		I have a feeling of being in nature and a strong sense of belonging (PI3)	0.900		
Pro-environmental behaviour intentions	Compliance Pro-environmental behaviour intentions	I will abide by the visitor code of conduct (CPEBI1)	0.959	KMO = 0.743 Sig. = 0.000	0.938
		I will abide by social ethics (CPEBI2)	0.958		
		I will respect local customs, cultural traditions and religious beliefs (CPEBI3)	0.916		
	Positive pro-environmental behaviour intentions	I will guide others to put their garbage in the box (PPEBI1)	0.818	KMO = 0.717 Sig. = 0.000	0.879
		I will warn and stop others from harming the environment (PPEBI2)	0.867		
		I will reflect the relevant environmental situation to the scenic spot or relevant departments (PPEBI3)	0.751		

Note: environmental knowledge perception (EKP); heritage genes perception (HGP); place dependence (PD); place identity (PI); compliance pro-environmental behaviour intentions (CPEBIs); positive pro-environmental behaviour intentions (PPEBIs).

**Table 4:** Test results of goodness-of-fit indices for SEM.

Fit Indices	CMIN/DF	RMSEA	AGFI	CFI	NFI	PGFI
Standard value	1–3	<0.08	>0.80	>0.90	>0.90	>0.5
Original model	2.925	0.079	0.808	0.922	0.887	0.774
Correction Model	2.432	0.068	0.835	0.942	0.906	0.667

**Table 5:** Reliability and validity test. *** *p* < 0.001.

Latent Variable	Items	The Standardised Factor Loadings	Parameter Significance Estimation				Items Reliability	Combination reliability	Average of Variance Extracted
		Std	Ustd	S.E	C.R	*p*	SMC	CR	AVE
HGP	HGP6	0.701	1				0.491	0.894	0.588
	HGP5	0.644	0.905	0.083	10.888	***	0.415		
	HGP4	0.655	1.159	0.107	10.837	***	0.429		
	HGP3	0.766	1.277	0.101	12.597	***	0.587		
	HGP2	0.897	1.361	0.096	14.245	***	0.805		
	HGP1	0.895	1.398	0.098	14.242	***	0.801		
EKP	EKP4	0.625	1				0.391	0.849	0.591
	EKP3	0.636	1.092	0.077	14.214	***	0.404		
	EKP2	0.917	1.236	0.104	11.913	***	0.841		
	EKP1	0.854	1.124	0.097	11.64	***	0.729		
PD	PD1	0.894	1				0.799	0.745	0.533
	PD2	0.85	1.014	0.057	17.883	***	0.723		
	PD3	0.28	1.256	0.265	4.74	***	0.078		
PI	PI1	0.854	1				0.729	0.858	0.668
	PI2	0.754	1.056	0.071	14.976	***	0.569		
	PI3	0.841	1.002	0.056	18.012	***	0.707		
CPEBIs	CPEBI1	0.954	1.000				0.910	0.941	0.842
	CPEBI2	0.954	0.969	0.028	34.183	***	0.910		
	CPEBI3	0.84	0.923	0.040	23.335	***	0.706		
PPEBIs	PPEBI1	0.869	1				0.755	0.888	0.726
	PPEBI2	0.913	1.095	0.056	19.623	***	0.834		
	PPEBI3	0.768	1.054	0.067	15.649	***	0.590		

**Table 6:** Differentiation validity test.

	AVE	EKP	HGP	PD	PI	CPEBIs	PPEBIs
EKP	0.591	0.769					
HGP	0.588	0.482	0.767				
PD	0.533	0.477	0.595	0.730			
PI	0.668	0.515	0.613	0.852	0.817		
CPEBIs	0.842	0.302	0.471	0.526	0.658	0.918	
PPEBIs	0.726	0.422	0.350	0.412	0.509	0.465	0.852

**Table 7:** Results of hypotheses test. ** *p* < 0.01, *** *p* < 0.001.

Hypothesised Relationship	Standardised Path Coefficient	T-Value	Test Results
H1: environmental knowledge perception → heritage genes perception	0.482 ***	6.574	Valid
H2: environmental knowledge perception → place dependence	0.248 ***	3.8	Valid
H3: environmental knowledge perception → place identity	0.106	2.03	Not valid
H4: environmental knowledge perception → compliance pro-environmental behaviour intentions	−0.075	−1.195	Not valid
H5: environmental knowledge perception → positive pro-environmental behaviour intentions	0.239 ***	3.394	Valid
H6: heritage genes perception → place dependence	0.475 ***	6.994	Valid
H7: heritage genes perception → place identity	0.132	2.263	Not valid
H8: heritage genes perception → compliance pro-environmental behaviour intentions	0.147	2.127	Not valid
H9: heritage genes perception → positive pro-environmental behaviour intentions	−0.020	−0.265	Not valid
H10: place dependence → place identity	0.723 ***	10.559	Valid
H11: place dependence → compliance pro-environmental behaviour intentions	−0.154	−1.161	Not valid
H12: place dependence → positive pro-environmental behaviour intentions	−0.076	−0.526	Not valid
H13: place identity → compliance pro-environmental behaviour intentions	0.738 ***	5.195	Valid
H14: place identity → positive pro-environmental behaviour intentions	0.303	1.782	Not valid
H15: compliance pro-environmental behaviour intentions → positive pro-environmental behaviour intentions	0.242 **	3.059	Valid

**Table 8:** Mediation effect results.

Action Path		Intermediary Type	Confidence Interval				Std.	Proportion of IE	Results
			Bias-Corrected 95%CI		Percentile 95% CI				
			Lower	Upper	Lower	Upper			
EKP→PD		OE	0.699	2.350	0.710	2.410	1.163	/	Partial mediating effect
		DE	0.200	1.307	0.213	1.360	0.603	/	
Path 1	EKP→HGP→PD	IE	0.233	1.387	0.226	1.344	0.560	0.481	
EKP→PI		OE	0.327	0.610	0.324	0.604	0.455	/	Full mediating effect
		DE	0.027	0.238	−0.010	0.203	0.119	/	
Path 2	EKP→PD→PI	IE 1	0.026	0.296	0.228	0.499	0.166	0.364	
Path 3	EKP→HGP→PD→ PI	IE 2	0.079	0.311	0.078	0.309	0.171	0.375	
EKP→CPEBIs		OE	0.102	0.363	0.098	0.353	0.211	/	Full mediating effect
		DE	−0.095	0.083	−0.103	0.076	−0.012	/	
Path 4	EKP→PD→PI→ CPEBIs	IE 1	0.041	0.203	0.046	0.207	0.117	0.556	
Path 5	EKP→HGP→PD→ PI→CPEBIs	IE 2	0.047	0.222	0.042	0.208	0.106	0.503	
EKP→PPEBIs		OE	0.274	0.569	0.273	0.566	0.402	/	Partial mediating effect
		DE	0.188	0.443	0.182	0.437	0.299	/	
Path 6	EKP→PD→PI→ CPEBIs→PPEBIs	IE 1	0.019	0.117	0.017	0.112	0.055	0.136	
Path 7	EKP→HGP→PD→ PI→CPEBIs→PPEBIs	IE 2	0.02	0.128	0.016	0.107	0.049	0.121	

Note: overall effect (OE); direct effect (DE); indirect effect (IE).

## Data Availability

The data are not publicly available due to institutional copyright and privacy issues. Requests to access the datasets should be directed to the email, renqingliu19@mails.ucas.ac.cn.
